# A prospective randomised control trial comparing functional with mechanical axis alignment in total knee arthroplasty: study protocol for an investigator initiated trial

**DOI:** 10.1186/s13063-021-05433-z

**Published:** 2021-08-09

**Authors:** Richard Steer, Beth Tippett, R. Nazim Khan, Dermot Collopy, Gavin Clark

**Affiliations:** 1 Gold Coast University Hospital, 1 Hospital Boulevard, Southport, Queensland Australia; 2 University of Queensland, St Lucia, Queensland Australia; 3 Perth Hip and Knee Clinic, 1 Wexford St, Subiaco, Western Australia Australia; 4 St John of God Hospital, Subiaco, Western Australia Australia; 5 University of Western Australia, 35 Stirling Hwy, Crawley, Western Australia Australia

**Keywords:** Functional alignment, Kinematic alignment, Mechanical alignment, Total knee arthroplasty, Clinical outcomes

## Abstract

**Background:**

A drive to improve functional outcomes for patients undergoing total knee arthroplasty (TKA) has led to alternative alignment being used. Functional alignment (FA) uses intraoperative soft tissue tension to determine the optimal position of the prosthesis within the patient’s soft tissue envelope. Angular limits for bone resections are followed to prevent long-term prosthesis failure. This study will use the aid of robotic assistance to plan and implement the final prosthesis position. This method has yet to be compared to the traditional mechanically aligned (MA) knee in a randomised trial.

**Methods:**

A blinded randomised control trial with 100 patients will be undertaken via Perth Hip and Knee Clinic. Fifty patients will undergo a MA TKA and fifty will undergo a FA TKA. Both alignment techniques will be balanced via computer-assisted navigation to assess prosthetic gaps, being achieved via the initial bony resection and further soft tissue releases as required to achieve satisfactory balance. The primary outcome will be the Forgotten Joint Score (FJS) 2 years after surgery, with secondary outcomes being other patient-reported outcome measures, clinical functional assessment, radiographic position and complications. Other data that will be collected will be patient demography (sex, age, level of activity) and medical information (grade of knee injury, any other relevant medical information). The linear statistical model will be fitted to the response (FJS), including all the other variables as covariates.

**Discussion:**

Many surgeons are utilising alternative alignment techniques with a goal of achieving better functional outcomes for their patients. Currently, MA TKA remains the gold standard with good outcomes and excellent longevity. There is no published RCTs comparing FA to MA yet and only two registered studies are planned or currently in progress. This study utilises a FA technique which differs from the two studies. This study will help determine if FA TKA has superior functional results for patients.

**Trial registration:**

This trial has been registered with the Australian New Zealand Clinical Trials Registry (ANZCTR) http://www.anzctr.org.au: U1111-1257-2291, registered 25th Jan 2021. It is also listed on www.clinicaltrials.gov: NCT04748510.

**Supplementary Information:**

The online version contains supplementary material available at 10.1186/s13063-021-05433-z.

## Administrative information

Note: the numbers in curly brackets in this protocol refer to SPIRIT checklist item numbers. The order of the items has been modified to group similar items (see http://www.equator-network.org/reporting-guidelines/spirit-2013-statement-defining-standard-protocol-items-for-clinical-trials/).

**Table:** 

Title {1}	A prospective randomised controlled trial comparing Functional with Mechanical Axis Alignment in total knee arthroplasty: study protocol for an investigator initiated trial
Trial registration {2a and 2b}.	Australia and New Zealand Clinical Trials Registry (ANZCTR): U1111-1257-2291 www.clinicaltrials.gov: NCT04748510
Protocol version {3}	Version 1.6, 4th March 2021
Funding {4}	Stryker (Kalamazoo, MI, USA)- Are providing VERASENSE sensor devices (Orthosensor Inc, Florida, USA), and funding for the arthrometers and dynamometers used in this study. St John of God Healthcare (Perth, WA, Australia)- providing funding support for a research physiotherapist and also covering the cost of insurance for the study.
Author details {5a}	Dr Richard Steer ^1,2,4^ Ms Beth Tippett ^3,4^ Dr R Nazim Khan ^4^ Mr Dermot Collopy ^3,4^ Mr Gavin Clark ^3,4,5^ 1 Gold Coast University Hospital- 1 Hospital Boulevard, Southport, Queensland, Australia 2 University of Queensland- St Lucia, Queensland, Australia 3 St John of God Hospital- Subiaco, Western Australia, Australia 4 Perth Hip and Knee Clinic- 1 Wexford St, Subiaco, Western Australia, Australia 5 University of Western Australia- 35 Stirling Hwy, Crawley, Western Australia, Australia
Name and contact information for the trial sponsor {5b}	St John of God Healthcare Level 1, 556 Wellington StreetPerth WA 6000Tel: + 61 8 6116 0000Fax: + 61 8 6116 0800Email: info@sjog.org.au
Role of sponsor {5c}	St John of God Healthcare has provided governance for this study including HREC oversight, insurance for the study along with financial support for a research physiotherapist

## Introduction

### Background and rationale {6a}

Total knee arthroplasty (TKA) is the most effective treatment for patients with symptomatic end-stage knee osteoarthritis that has not responded to appropriate non-operative management. The aims of TKA are to provide pain relief and improve function. Accuracy of limb alignment, implant positioning and soft tissue balance after TKA are important prognostic factors that affect postoperative clinical outcomes and long-term implant survivorship [1, 2]. Published literature has shown between 80 and 90% of patients are satisfied following this procedure [3–6].

Native knee alignment and joint line obliquity is known to be variable in the population [7]. To this end, there are much debated theories of postoperative alignment after TKA. A mechanically aligned (MA) TKA has been shown to have good long-term survivorship [8]. In total knee arthroplasty with MA, the aim is to achieve neutral limb alignment in the coronal plane. This is achieved by cutting the distal femur and proximal tibia perpendicular to the mechanical axis of the limb. A compensatory external rotation of the femoral component is made to account for the loss of the ‘normal’ 3° varus joint line. This is to optimise longevity of TKA and prevent early failure. A recent study by MacDessi et al. [9] looking at the coronal plane alignment along with joint line obliquity found that only 15.4% of normal knees and 14.6% of arthritic knees have a mechanically neutral alignment and joint line. Therefore, in approximately 85% of patients, a mechanically neutral joint replacement is not restoring their constitutional alignment. There have also been variations in mechanical alignment including adjusted MA, leaving some residual joint line obliquity rather a full correction to mechanically neutral [10]. This has shown promising outcome improvements [11].

Total knee arthroplasty with kinematic alignment (KA) aims to restore the patient’s own pre-arthritic knee anatomy (constitutional knee anatomy) with more natural alignment and preservation of native function [12]. This is done via a variety of methods, based on bone/cartilage landmarks to determine the bone resections. A restricted kinematic alignment option has also been proposed with coronal plane limits on the femoral and tibial resections to 5° and a hip-knee-ankle arthrimetic alignment within 3° of neutral [13, 14].

Prospective studies comparing functional outcomes between the MA and KA groups have been performed with varying results, some favouring KA and others suggesting no difference. The main limitation of these studies has been the inability to accurately measure the desired deviation from neutral alignment as well as achieving the implant position to a high degree of accuracy. There is a paucity of studies using standardised techniques for intraoperative alignment and limited data relating these findings to clinical outcomes with long-term follow-up [15–21].

Enthusiasts debate these theories but it is yet to be established whether one is superior to the other.

An issue with both of these alignment theories is that they are purely related to bony anatomy. Neither considers soft tissue tension or balance before the bony cuts are made. Once the bony cuts are made, the soft tissue balance is assessed and ligaments released until the TKA is balanced to the surgeon’s satisfaction. In a study by MacDessi et al. [20] if either MA or restricted KA methods are followed, soft tissue releases or bony recuts are required to achieve a balanced TKA, MA being unbalanced more often.

The accuracy of soft tissue balancing is surgeon dependent, and there is evidence that even experienced knee surgeons are poor at manually determining if a knee is balanced [22]. This becomes particularly important when considering over half of these procedures throughout the world are performed by surgeons who undertake less than 25 TKA per year [23].

Robotically assisted TKA (RATKA) with the Mako robotic arm (Stryker, Florida, USA) has been shown to improve accuracy in implant positioning and early functional rehabilitation; however, the medium to long-term benefits are yet to be proven [24]. Planning software associated with the Mako robotic arm has allowed the development of a pre-resection balancing technique. This enables assessment of soft tissue laxity and adjustment of the initial plan to achieve balanced soft tissue with alteration of component alignment. Once the knee has been virtually balanced on the planning software, robotic arm-assisted surgery is undertaken to accurately replicate the plan resulting in a balanced TKA [25].

Functional alignment (FA) has the goal of restoring constitutional joint line obliquity using the native bony anatomy along with soft tissue tension to determine the optimal prosthesis alignment. Functional alignment ideally minimises the need for soft tissue release. As the collateral ligaments should not contract through the disease process of osteoarthritis, re-tensioning these ligaments following the removal of osteophytes should act as a surrogate of individual limb alignment. A preoperative CT scan is used with planning software to place the TKA prosthesis in a ‘resurfacing’ position based on bony landmarks, similar to that aimed in a KA TKA. Intraoperatively, prior to any bony resections, the planning software is used to assess soft tissue balance with the planned prosthesis position and then the plan adjusted accordingly. Component positioning limits (discussed in surgical technique) are used so as not to place the prosthesis in a position deemed to be at risk for migration and implant failure. A FA TKA technique has been shown to produce a well-balanced knee as assessed with VERASENSE (Orthosensor Inc, Florida, USA) [25]. A paper by Oussedik et al. [26] discussing the various knee alignment techniques currently in use, lists FA as having a tibial coronal plane position of between 0 and 3° varus. They accept that further study may change this limit including an overall hip-knee-ankle alignment of neutral ± 3°. Greater tolerances in component positioning are already in practice, including tibial component varus alignment of up to 6°. This study will utilise those limits (detailed further in section ‘Intervention description {11a}’) to assess FA TKR.

Although the overall limb alignment in this technique is independent of the initial plan, the individual component position and joint line obliquity will vary depending on whether the knee was planned with a mechanical axis starting point or a kinematic axis alignment starting point. Assessment of the Perth Hip and Knee Registry data would suggest on average a 2° difference in both tibial and femoral coronal alignment, femoral rotation and difference of joint line obliquity despite having the same hip-knee-ankle angle (HKA) coronal plane alignment.

The need for soft tissue release to balance the knee in both MA and KA alignment makes them unsuitable for a computer-enabled planning and balancing algorithm. This is due to variation in surgical skill and the difficulty in standardising soft tissue releases. Functional alignment is well suited to automated algorithms as the surgical steps to balance the knee (bony cuts) would be robot assisted and therefore have an in-built quality control. The development of computer algorithms to plan and balance the knee arthroplasty could potentially improve the overall quality of TKA performed.

Limits are placed in functional alignment to prevent the severely arthritic knee with attenuated ligaments being placed in extremes of alignment. Understanding the ‘safe zone’ for functional alignment is important for patient selection, implant choice, extent of intraoperative deformity correction and long-term follow-up. Furthermore, some disease pathology such as bone tumours, previous trauma and congenital deformities may be present in conjunction with the arthritic knee and will have altered the native alignment of the limb. In such cases, using functional alignment to reproduce the altered anatomical alignment and mechanical function may have a detrimental effect on knee mechanics and wear.

### Current evidence

There are no published prospective studies looking at functional alignment. There is an RCT currently underway at University College London Hospital [27] and another RCT comparing a balanced MA TKR with FA TKR in New Zealand which was scheduled to begin recruitment in July 2020 [28]. The authors are also aware of another multi-centre study due to begin comparing MA and KA in both RAS and computer-navigated arthroplasty, beginning in 2021 in Australia [29]. This study will differ from the other studies in a different algorithm being used to achieve a functionally aligned TKR. The results of the various trials may help guide which method of achieving a FA TKR has the best outcomes.

### Need for a trial

The need for studies to define potential benefits of alternative alignment requires accurate execution of plans. There is a need for high-quality evidence on the clinical and radiological benefits of functionally aligned TKA. This study would investigate if there are any superior outcomes to be obtained from functional alignment. Clinical and functional outcomes should also be correlated to longer-term outcomes to better establish the ‘safe zone’ for functional alignment.

Currently, the vast majority of TKA throughout the world is undertaken utilising MA alignment. Any change to a newer technique from the current ‘gold standard’ would need to be justified by improved clinical outcomes as there is no long-term longevity data for functional alignment. Both surgeons and patients are only able to consider the relative risks and benefits of this technique once they are defined by sound scientific evidence. This study will contribute to the body of this evidence.

### Objectives {7}

The overall aims of this prospective, randomised double-blinded controlled trial are to compare functional, clinical and radiological outcomes in FA TKA versus MA TKA. Patients undergoing MA TKA will form the control group and those undergoing FA TKA will form the investigation group. A superiority design will be used to evaluate whether FA TKA provides superior outcomes compared to MA TKA. Primary and secondary objectives will be used to quantify and draw inferences on differences in the efficacy of treatment between the two groups. To ensure accuracy of planning and implantation robotic arm-assisted surgery will be utilised in both groups (Stryker MAKO, Fort Lauderdale, FL). Standardised postoperative care will be undertaken to ensure the only difference between groups is alignment of implants.

### Primary outcomes

The aim of this study is to compare clinical outcomes between MA TKA (control group) and FA TKA (investigation group) at 2 years following surgery. The null hypothesis is that there is no difference in functional scores at 2 years following surgery between patients undergoing MA TKA versus FA TKA. The primary outcome measure for this study is the Forgotten Joint Score (FJS) at 2 years after surgery. The hypothesis is that the FJS obtained at 2 years after surgery in patients undergoing FA TKA are not achievable using MA TKA.

### Secondary outcomes

#### PROMs

- Further patient-reported outcome measures (PROMs) will be used to further assess clinical outcomes including Oxford Knee Score (OKS), Knee Injury and Osteoarthritis Outcome Score for Joint Replacement (KOOS JR), VAS Pain and Kujala scores preoperatively and postoperatively at 3 months, 1 year and 2 years.
- Health-related quality of life will be measured using European Quality of Life questionnaire with five dimensions for adults (EQ-5D-5L) preoperatively and postoperatively at 3 months, 1 year and 2 years.
- Range of movement (°) in knee joint during inpatient admission and postoperatively at 6 weeks, 3 months, 1 year and 2 years.

#### Alignment measures

To determine lower limb alignment achieved with both alignment techniques. Lower limb alignment is assessed using intraoperative optical verification along with standing long leg radiographs performed postoperatively at 3 months. Measurements of the hip-knee-ankle angle (HKA), medial proximal tibial angle (MPTA) and lateral distal femoral angle (LDFA). Also evidence of imbalance with implant lift off will be measured.

#### Analgesia

To determine if there are any differences in analgesic requirements based on alignment method used, analgesia requirements during inpatient admission and postoperatively at 6 weeks, 3 months, 1 year and 2 years will be measured.

#### Balance and stability

- To determine whether alignment method utilised has an effect on the sagittal stability of the TKA, assessment of sagittal stability at pre-op, 3 months, 1 year and 2 years will be undertaken with an arthrometer ‘Lachmeter’ (see additional file 1).
- To determine whether alignment method utilised has an effect on functional tests, this will be assessed by a combination of;
- Maximum voluntary isometric force using a dynamometer at pre-op, 3 months, 1 year and 2 years.
- Sit to stand values as measure of function at pre-op, 3 months, 1 year and 2 years.
- Intraoperative balance achieved with both alignment techniques. Surgeon-blinded measurement of intraoperative balance achieved with VERASENSE sensor (Orthosensor Inc, Florida, USA)—smaller cohort of approximately 60 participants.
- To determine if there is a difference in knee kinematics between the two techniques, measurement of knee kinematics was done with VERASENSE sensor (Orthosensor Inc, Florida, USA) to assess presence or absence of medial pivot—smaller cohort of approximately 60 participants.

### Trial design {8}

This study is a prospective, single-centre, randomised, double-blinded, controlled study. Patients undergoing MA TKA will form the control group, and those undergoing the FA TKA will form the investigation group.

## Methods: participants, interventions and outcomes

### Study setting {9}

The study base will be Perth Hip and Knee Clinic, 1/1 Wexford St, Subiaco 6008, WA, Australia. All patients will have surgery and inpatient stay at St John of God Subiaco Hospital, Perth, WA, Australia. Recruitment and follow-up will be at either Perth Hip and Knee Clinic (Subiaco or Murdoch rooms) or Midland Orthopaedics (Suite 11 St John of God Midland Hospital, Clayton Rd. Midland).

### Eligibility criteria {10}

#### Inclusion criteria

- Patient has symptomatic knee osteoarthritis requiring primary TKA
- Patient and surgeon are in agreement that TKA is the most appropriate treatment
- Patient is fit for surgical intervention following review by surgeon
- Patient is between 45 and 75 years of age at time of surgery, computer literate and able to complete patient-reported outcome measures independently
- Patient must be capable of giving informed consent and agree to comply with the postoperative review programme
- Patient must be a permanent resident in an area accessible to the study site
- Patient must have sufficient postoperative mobility to attend follow-up clinics and allow for radiographs to be taken
- Patient has tried non-pharmacologic therapy’s including patient education, self-management programmes, aerobic exercise, weight loss, physiotherapy and occupational therapy
- Patient has tried appropriate pharmacologic therapies including regular paracetamol and NSAIDs if appropriate

#### Exclusion criteria

- Patient is not suitable for routine primary TKA, e.g. patient has ligament deficiency that requires a constrained prosthesis
- Intraoperative requirement for a more constrained implant
- Knee found to be PCL deficient and require a posteriorly stabilised prosthesis. These patients will be still included in the study, but analysed with an intention to treat principal.
- Patient has bone loss that requires augmentation
- Patient requires revision surgery following previously failed correctional osteotomy or ipsilateral TKA (e.g. post high tibial or distal femoral osteotomy)
- Patient requires a polyethylene inset of 16 mm or greater
- Patient is immobile or has another neurological condition affecting musculoskeletal function
- Patient is less than 44 years of age or greater than 76 years of age
- Patient is a compensable patient, i.e. worker’s compensation claim or motor vehicle accident
- Patient is already enrolled on another concurrent clinical trial
- Patient is unable or unwilling to sign the informed consent form specific to this study
- Patient is unable to attend the follow-up programme
- Patient is non-resident in local area or expected to leave the catchment area postoperatively
- Patients who lack capacity to provide consent, or the ability to understand the study protocol due to a cognitive condition (e.g. dementia)
- Patient is unable to communicate effectively in English

### Who will take informed consent? And recruitment {26a}

Participants will be assessed by the treating orthopaedic consultant surgeon. If the patient meets all of the inclusion criteria and none of the exclusion criteria, and expresses an interest to participate in the study, he will be provided with a patient information sheet. This provides details about the study, treatment, follow-up and contact details for further information. Details of those patients expressing an interest to participate in the study will be recorded in the patient contact sheet, which will be a password-protected excel document that only investigators and research physiotherapist will have access to. One week after this outpatient consultation, the research physiotherapist will telephone the patient to answer any additional queries and confirm whether or not the patient would like to participate in the study. If the patient agrees to participate in the study, the orthopaedic fellow will randomise the patient into one of the two treatment groups. Patients allocated to MA TKA will form the ‘control group’ whilst those allocated to FA TKA will form the ‘investigation group’.

The anticipated length of time between initial consultation and obtaining informed consent for inclusion into the study is at least 1 week. This method provides time for potential participants to consider the trial and ask questions before written consent for participation is requested.

Written informed consent for both the operative procedure and inclusion into the study will be signed at the preadmission appointment by the operating surgeon.

If the patient initially agrees to participate in the study and then changes his/her mind at a later stage, they are free to do so without any compromise to their further care. If this occurs before obtaining informed consent, then the patient’s decision will be relayed to the Operating Surgeon who will discuss suitable options directly with the patient, and organise postoperative follow-up care as per all routine (non-study) patients undergoing TKA at Perth Hip & Knee Clinic. If the patient agrees to participate in the study and then declines further inclusion after surgery has been performed, then the patient’s follow-up care will be arranged as per routine TKA follow-up. Following randomisation and informed consent, baseline information will be recorded and documented in the baseline investigator form.

Participants will not receive any preferential treatment or payment for taking part in the study.

All patients included into this study are free to withdraw from the study at any time without compromise to their future treatment. On withdrawal, patients will revert to the standard follow-up regimen for routine TKAs at the study site. The end of study form will be completed and the reason for withdrawal documented. This form will also be completed if the patient is lost to follow-up or dies during the course of the study.

Enrolled patients will be withdrawn from the study if:

- The patient withdraws consent for participation in the study
- The patient is no longer able to comply with study instructions, attend scheduled appointments or complete questionnaires
- The patient undergoes implant revision

Data to the point of withdrawal will be used for analysis.

### Additional consent provisions for collection and use of participant data and biological specimens {26b}

Incorporated into the consent process for participation listed in section ‘Who will take informed consent? And recruitment {26a}’.

### Interventions

#### Explanation for the choice of comparators {6b}

MA TKA has been the standard of treatment for alignment for TKA, but not without lack of satisfactory functional outcomes in a subset of patients. The use of FA TKA has been proposed to offer better functional outcomes for the patient. This has yet to be proven with an RCT comparing functional outcomes of FA TKA with the current gold standard of MA TKA.

#### Intervention description {11a}

##### Surgical intervention: MA vs. FA TKA

All patients undergoing TKA will undergo preoperative CT scan of the leg to establish the extent of the disease process, determine bone resection and plan implant sizing and positioning. The preoperative CT scan will be used to create individualised plans for achieving mechanical and kinematic alignment and stored within the robotic programme that is used during the operative procedure. This will ensure that all implants and equipment for achieving either mechanical alignment or functional alignment are ready and available for use in theatre. These plans will provide the initial point from which functional alignment will be achieved.

Following informed consent and randomisation into one of the two treatment groups, patients will undergo robotic arm-assisted TKA by one of the participating surgeons. Surgery in both groups will be undertaken through the standard anteromedial arthrotomy with positioning of reference pins in the femur and tibia for registration of the hip centre, ankle position and limb alignment. Cruciate retaining technique will be used in both groups. Interoperative requirement for a more constrained implant will result in exclusion from the study.

In MA TKA, tibial and femoral osteotomies in the coronal plane will be planned perpendicular to the tibial and femoral mechanical axes respectively to achieve neutral overall alignment. Soft tissue balance will be assessed and minor adjustments to bony alignment made to balance the knees with a maximal adjustment of 2° valgus and 2° varus of coronal alignment from neutral. Femoral rotation will be planned to surgical epicondylar axis and adjustments to rotation made to allow equal flexion and extension balance (to within 1 mm). If balance cannot be achieved within these boundaries, then soft tissue release will be undertaken.

In the sagittal plane, 0–3° of posterior tibial slope and 0–5° of femoral component flexion will be used to optimise implant sizing whilst preventing notching. In the axial plane, the tibial component aligned to Akagi’s line, which connects the medial border of the patellar tendon attachment to the middle of the posterior cruciate ligament attachment on the tibia [30].

In FA TKA, femoral and tibial osteotomies will be planned for equal bony resections from the femoral condyles to replicate the patient’s anatomy. In the coronal plane, the distal femoral resection will be 6.5 mm from the subchondral bone of both medial and lateral condyles, with compensation for wear by adjusting the resection by 1–3 mm. In the proximal tibia, there will be 7 mm of resection from the subchondral bone from both the medial and lateral tibial plateau. In the sagittal plane, resection angle will be determined intraoperatively to closely match the native femoral flexion and tibial slope. In the axial plane, posterior femoral resection will be 6.5 mm from the subchondral bone of both medial and lateral posterior condyles.

Tibial rotation will be aligned to Akagi’s line [30]. Adjustments will be made to bony alignment to balance soft tissues within the boundaries of 6° varus and 3° valgus hip-knee-ankle angle (HKA) alignment. Femoral component alignment will be limited to 6° of valgus and 3° of varus in the coronal plane. Tibial alignment will be limited to 6° of varus and 3° of valgus in the coronal plane. Combined flexion of the components will be limited to 10° of flexion. Only if balance cannot be achieved within these boundaries will soft tissue release be undertaken.

In both groups, polyethylene thickness will be selected to maximise range of motion whilst avoiding hyperextension and ligament laxity. Tibial depth will be adjusted to maintain insert thickness between 9 and 14 mm. Any TKA requiring a 16 mm or greater polyethylene inset thickness will be excluded from the study. Use of polyethylene of 16 mm or greater is usually associated with severe deformity with ligamentous laxity, making it a relative contraindication for functional alignment, as this technique relies on the intact collateral ligaments to guide final alignment. A CR polyethylene will be used for all cases.

Patients in both groups will undergo the same inpatient and outpatient postoperative rehabilitation programme. Intraoperative data will be recorded using the surgical data form. The only difference between the two treatment groups is that the control group will undergo MA TKA and the investigation group will undergo FA TKA.

##### Description of the device

The Stryker Triathlon (Stryker, Kalamazoo, MI, USA) cruciate retaining knee system with patellar resurfacing will be used in both groups. The femoral component will be un-cemented, and patella and tibial components will be cemented. This implant and its surgical instruments are already in routine use for TKA at St John of God Subiaco Hospital. The surgical team are fully trained and experienced with the use of the instruments and surgical equipment for these implants.

#### Criteria for discontinuing or modifying allocated interventions {11b}

There will be no formal criteria for discontinuing or modifying an allocated intervention. The prosthesis and use of RATKA is already approved and utilised daily both in Australia and internationally. The two alignment techniques are already currently used for TKA. The primary outcome for the study will occur after recruitment has been completed.

#### Strategies to improve adherence to interventions {11c}

As the intervention will be a one-off event, adherence will be centred around the participants receiving the correct arm of the study they are allocated to. Having only two surgeons performing the procedure and one orthopaedic fellow randomising the participants into groups maximises the likelihood of adherence to the correct intervention.

#### Relevant concomitant care permitted or prohibited during the trial {11d}

Patients in both groups will undergo the same inpatient and outpatient postoperative rehabilitation programme. Intraoperative data will be recorded using the surgical data form. The only difference between the two treatment groups is that the control group will undergo MA TKA and the investigation group will undergo FA TKA.

#### Provisions for post-trial care {30}

All participants will continue usual follow-up and care with their treating surgeon, the investigators, following completion of the trial. Any adverse outcomes as a result of TKA will be managed as per usual health care practice of those not involved in the study.

#### Outcomes {12}

The FJS, EQ5D-5 L, OKS, VAS Pain, Likert scale, KOOS JR and Kujala anterior knee pain scores are validated tools for the clinical assessment of patients after knee arthroplasty [31, 32]. Each of the outcome scores is completed preoperatively and then at regular intervals during follow-up (section ‘Participant timeline {13}’, Table 1) Timelines for radiographic imaging can be seen in Table 2. Analysis will be made between preoperative mean data (if normally distributed, otherwise median data will be used) to 2-year outcomes, along with comparison between groups. Routine clinical measures of height, weight, range of movement and pain description will be taken.

**Table 1:** Timelines for clinical data collection in all study patients

	Preoperative	Discharge	6 weeks	3 months	1 year	2 years
Patient demographics	X					
Patient medical history	X					
Operation details		X				
Clinical history and PROMS (FJS, EQ5D-5L, OKS, VAS pain, KOOS Jr, Kujala Anterior Knee Pain, Likert scale)	X		X (FJS and VAS only)	X	X	X
Functional Examination (Lachmeter testing, Range of Movement, Hand Held Dynamometer, 30s STS test)	X	X (range only)	X (range only)	X	X	X
Adverse Events	At occurrences through study period

**Table 2:** Timelines for radiological data collection in all study patients. Assessment windows will be as follows: 6-week review (± 1 week), 3-month review ((± 2 weeks), 12-month review (± 2 months), 2-year review (± 2 months)

	Preoperative	Discharge	6 weeks	3 months	1 year	2 years
Plain knee joint radiographs	X	X			X	X
Plain long-leg radiographs	X			X		
CT Knee joint	X					

To test endurance and strength, a 30-s sit to stand test will be measured. This test asks the participant to stand up / sit down from a standardised chair height within 30 s. To measure the AP stability of the knee [33], a Lachmeter reading will also be recorded at the postoperative intervals, and to measure strength, a dynamometer test will also be performed [34–36]. More detail of these tests can be found in additional file 1.

#### Participant timeline {13}

Patients will be recruited from the private rooms at Perth Hip & Knee clinic. Based on the volume of TKAs performed and recruitment rates from previous studies within clinic, patients are expected to be recruited at a rate of 10 patients per month. The recruitment process will therefore take approximately 10–12 months from the start of the study. From the date of the operation, each patient will be followed up for 24 months. A further 6 months will be required for data collection, analysis and dissemination of findings. The total duration of the study will therefore be 40 months. Recruitment is planned to begin in April 2021.

#### Sample size {14}

In total, 100 patients will be enrolled in a 1:1 ratio between the two treatment groups. This will ensure that the minimum of 90 patients required to answer the study question are followed up for the duration of the study. The enrolment goal is to have at least 45 patients in each of the two treatment groups completing the study.

#### Power / sample size calculation

For the primary outcome measure, functional outcome as assessed using the FJS score at 2 years following Mako arm-assisted TKA. Using data from our initial cohort recording functional outcomes, the mean FJS score at 1 year in the MA TKA was 59 (SD 6) and in the FA TKA was 75 (SD 8). It is assumed that MA results will be no better than FA results. The study was powered to demonstrate a 12-point difference in the Forgotten Joint Score. Whilst the minimal important difference (MID) has been reported with different values in the literature, Holtz et al. [37] calculated an MID at 2 years, most closely representing our study. A recent study published by Clement et al. [38] found the mean clinically important difference to be 13.7; this result was based on results at 6 months following surgery.

Using a one tailed analysis (assuming superior results with the FA), an alpha value of 0.05 and power of 0.80, and accounting for expected dropout rate of 10%, this study will need 100 patients to answer the study question. Note that a more careful assessment of power and sample size for linear mixed effects model requires assumptions on the other covariates in the model and simulations. Such modelling is not expected to provide any reduction in power, so we have chosen to avoid this at the moment.

#### Recruitment {15}

Refer to section ‘Who will take informed consent? And recruitment {26a}’.

### Assignment of interventions: allocation

#### Sequence generation {16a}

The system for randomisation will be the same throughout the study period and must be strictly adhered to. The following method will be used to allocate a trial patient to either the MA TKA (‘control group’) or to the FA TKA (‘investigation group’).

Randomisation will be carried out using a blocked effect. This method is designed to randomise subjects into two groups that result in equal sample sizes over time. The blocks will be small (*n* = 4) and balanced within the predetermined group assignments, which will keep the number of subjects in each group similar at all times. There will be no stratification factors involved in the randomisation as randomisation will occur before the trial starts. Using a randomisation website (www.random.org), a random number (between 1 and 100,000) will be generated. This will form the ‘seed’ number for the blocked randomisation process. Using a randomisation website (www.sealedenvelope.com) the randomisation list will be created. Patients will be allocated in a sequential order of consent, strictly adhering to the allocation of groups. Screenshots of the randomisation process and seed number will be taken throughout, and held from the CI to minimise randomisation bias.

#### Concealment mechanism {16b}

The orthopaedic fellow will then privately communicate to the CI the allocated group, to enable alignment and templating planning to be performed using the MAKO software. All patients and clinical staff recording the postoperative clinical outcomes of interest will remain blinded to minimise performance and detection bias.

#### Implementation {16c}

Randomisation and allocation to groups will be performed by the orthopaedic fellow. They will be responsible for communicating the group allocation to the investigating surgeons just prior to surgery for surgical planning.

### Assignment of interventions: blinding

#### Who will be blinded {17a}

The participants and physiotherapists collecting outcome measures both before and after surgery will be blinded to the treatment group allocation. The investigators will be unblinded prior to surgery in order for an appropriate treatment plan to be implemented.

#### Procedure for unblinding if needed {17b}

There should be no reason during the study in which unblinding is required. There is no increased risk of harm associated with either group allocation to the patient, which will necessitate unblinding to occur.

### Data collection and management

#### Plans for assessment and collection of outcomes {18a}

Clinical assessments and outcomes will be undertaken as the patient timeline (section ‘Participant timeline {13}’). All investigators will undertake training in the clinical assessment to ensure standardisation of assessments.

#### Plans to promote participant retention and complete follow-up {18b}

The study will use participants who live locally and under the age of 76 and will aid in the participant retention. All participants will be aware of the 2-year follow-up prior to agreeing to participate in the study, therefore decreasing the likelihood of dropout.

### Data management {19}

The principle of Good Clinical Practice will be adhered to throughout with the research team responsible for its own regular internal audit for quality, recruitment goals and results targets. This will be in the form of monthly research meetings for those involved in the trial. The investigator will designate one or more appropriately trained and qualified individuals to monitor the progress of the clinical study. As per section 2.1.1 of the NHMRC Code, all clinical trial research data will be retained for a minimum of 15 years from the date of publication or 5 years following the completion of the research.

The Chief Investigator will review and provide assurances of the training and experience of all staff working on this study. Appropriate training records will be maintained in the study files. All personnel working on this study will have completed the Guideline for Good Clinical Practice ICH E6(R2) Qualification.

All case report forms (CRFs) must be completed and signed by staff that are listed on the site staff delegation log and authorised by the CI/ PI to perform this duty. The CI/PI is responsible for the accuracy of all data reported in the CRF.

Data required according to this protocol are to be recorded on the CRFs as soon as possible. Patients will be identifiable with a unique study number. Only the research physiotherapist will have the key to identify individual patients. All CRFs must be legible and completed in black ink. Any necessary corrections are to be made by drawing a single line through the incorrect entry and writing the revision, and must be initialed and dated by the investigator or his or her representative. Data are not to be obliterated by blacking out, using correction fluid or by erasing the original entry. Any documents related to the study must be archived directly at the study site. These documents include listings that identify study subjects, research group allocated to each study subject, consent forms and all completed CRFs. All consent forms and CRFs will be stored by the CI/PI investigator in a locked filing cabinet in a dedicated locked research office. This office has key access with monitored security. Patient data will be logged electronically using each patient’s unique identification number with Socrates computer software on an encrypted, password-protected research computer on the Perth Hip and Knee Clinic network. This computer is located within a dedicated lockable research office within Perth Hip and Knee Clinic, Subiaco, Australia.

### Confidentiality {27}

See ‘ Data management {19}’.

### Plans for collection, laboratory evaluation and storage of biological specimens for genetic or molecular analysis in this trial/future use {33}

There will be no specimens collected during this study.

### Statistical methods

#### Statistical methods for primary and secondary outcomes {20a}

##### Primary endpoint

The analysis of the per-protocol population will be considered the primary analysis. Data exploration (numerical and graphical) will precede statistical modelling. A linear mixed effects statistical model will be fitted to the data with FJS score as response and the other variables (demographic and treatment) to assess any difference in mean FJS scores between the MA TKA and FA TKA groups after adjusting for the effects of the other covariates. Note that several measurements will be made on each patient over time, so an appropriate correlation structure will also be fitted into the model.

##### Secondary endpoints

An appropriate linear statistical model will be fitted to the secondary outcomes, depending on the outcome. For data with a longitudinal structure, mixed effects linear model with an appropriate correction structure will be fitted [39].

### Intention-to-treat population

The intention-to-treat (ITT) population is defined as all randomised patients assigned to either the MA TKA or FA TKA group, regardless of adherence with the entry criteria, regardless of the treatment they actually received, and regardless of subsequent withdrawal from treatment or deviations from the protocol. In the event that MA is converted to FA or vice versa intraoperatively, analysis will be performed using the ITT population and the treatment actually received by the patients. Intraoperative conversion from one method to another will however, be documented and presented/published as part of the study.

In the event that there are errors in the randomisation assignment, the analysis will be performed using the assigned treatment, not the treatment that the patient actually received. Any patient terminated early from the clinical trial will be included in the ITT population. All attempts will be made to collect complete follow-up evaluations for these patients despite study exit. These patients will be included in the analysis as the statistical model will allow for missing data at some follow-up time points.

### Per-protocol population

The per-protocol population is defined as all patients who are randomised to MA TKA or FA TKA and complete the study according to the protocol.

In the event that there are errors in the randomisation assignment, the analysis will be performed using the treatment that the patient actually received, not the assigned treatment. Patients will be considered protocol violators if they do not meet the eligibility criteria as outlined in the protocol. Other reasons to be considered a protocol violator include, but are not limited to, protocol violations, and any actions that compromise the effectiveness of the treatment, such as receiving a secondary treatment. Protocol violators will not be considered as part of the per-protocol population and will be listed separately with the reason for their exclusion from the per-protocol population.

### Interim analyses {21b}

There will be no interim analysis undertaken.

### Methods for additional analyses (e.g. subgroup analyses) {20b}

There will be a smaller subset within each group where a VERASENSE sensor (Orthosensor Inc, Florida, USA) will be used intraoperatively to assess for balance and symmetry of contact points. The orthopaedic surgeon will be blinded to this result.

### Methods in analysis to handle protocol non-adherence and any statistical methods to handle missing data {20c}

See section ‘Statistical methods for primary and secondary outcomes {20a}’.

### Plans to give access to the full protocol, participant level-data and statistical code {31c}

The full protocol or collected data will not be granted to the public. Participants will be provided with a lay summary of the research findings after completion of the study.

### Oversight and monitoring

#### Composition of the coordinating Centre and trial steering committee {5d}

The study base will be Perth Hip and Knee Clinic, 1/1 Wexford St, Subiaco 6008, WA. All patients will have surgery and their inpatient stay will be at St John of God Subiaco Hospital with recruitment and follow-up at either Perth Hip and Knee Clinic (Subiaco or Murdoch rooms) or Midland Orthopaedics (Suite 11 St John of God Midland Hospital, Clayton Rd. Midland). All confidential study information will be stored on designated password-protected research computers and assigned research offices at Perth Hip and Knee Clinic.

The trial steering committee will include the chief investigator, co-investigator, a research physiotherapist and orthopaedic clinical fellow for Perth Hip and Knee Clinic. They will meet on a fortnightly basis.

The study protocol has been reviewed by two external reviewers prior to HREC approval and trial registration.

#### Composition of the data monitoring committee, its role and reporting structure {21a}

No data analysis will not occur until completion of the study. However, an independent data safety monitor (IDSM) has been appointed for the study—Professor David Wood, University of Western Australia, Western Australia, Australia.

### Adverse event reporting and harms {22}

**Fig. 1:**
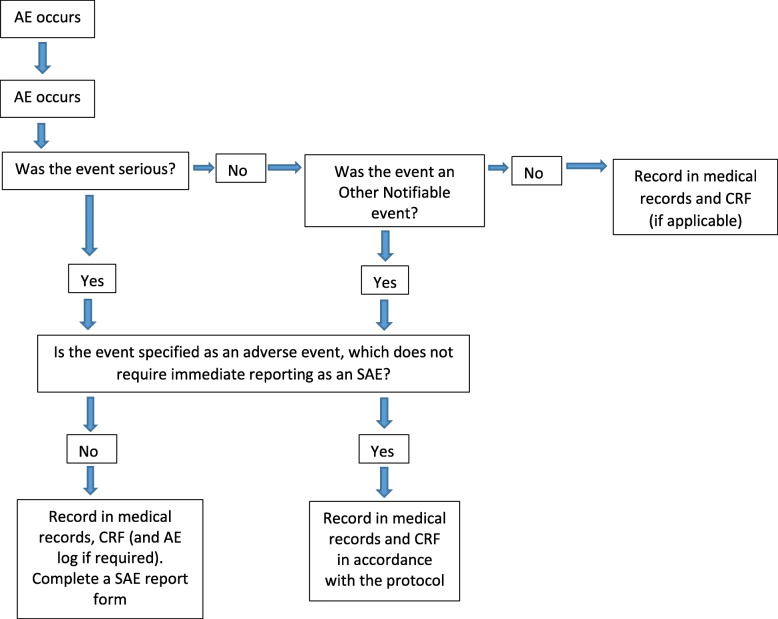
Flow diagram for reporting serious adverse events

All serious adverse events will be recorded in the medical records and the CRF, and the sites AE log. All serious adverse events (SAEs) must be recorded on a serious adverse event form (Fig. 1). The Principal Investigator will complete the SAE form and the form will be emailed to the SJOG HREC Committee within five working days of becoming aware of the event. The Chief Investigator will respond to any SAE queries raised by the primary HREC as soon as possible. Where the event is unexpected and thought to be related to the procedure, this must be reported by the Investigator to the Therapeutic Goods Administration via the Incident Reporting and Investigation Scheme within 15 days.

### Reporting incidents

#### Protocol deviations and notification of protocol violations

A deviation is usually an unintended departure from the expected conduct of the study protocol, which does not need to be reported to the TGA. The principal investigator will monitor protocol deviations.

A protocol violation is a breach which is likely to effect to a significant degree

- The safety or physical or mental integrity of the participants of the study; or
- The scientific value of the study.

The Chief investigator and IDSM will be notified immediately of any case where the above definition applies during the study conduct phase.

#### Reporting incidents involving a medical device

Adverse device effects (complications) are defined as any of the following:

- Any device component failure (e.g. excessive migration of the implant or failure otherwise).
- Local complications arising from use of the TKA implants to include osteolysis, inflammation, local tissue reaction and periprosthetic fracture
- Bone fracture during implantation
- Nerve damage arising from implant placement (as evidenced immediate postoperative by motor and/or unexpected sensory deficit)
- Large vessel damage arising during surgery (with large blood loss, i.e. > 1500 ml)
- Prosthetic joint infection
- Surgical site infection
- Loosening of prosthetic components
- Other adverse events that are deemed device related and serious

All serious adverse events, life-threatening problems or deaths that occur during or following the use of the devices during the study should be fully documented in the research record by the Chief Investigator including the onset date, complete description of the event, severity, duration, action taken and outcome. The event should be documented at the appropriate interval case report form. The Chief Investigator will be responsible for notifying the reviewing Research Ethics Committee, of any unanticipated adverse events according to local regulations. The Chief Investigator will record all non-serious adverse events on the appropriate case report form.

Some adverse events may lead to subsequent surgical intervention. The surgical intervention should be reported separately from the presentation of the other adverse event. For example, if the adverse event is reported at the 3-month visit and a revision subsequently occurs after the 3-month visit, the revision should be reported in the next follow-up visit.

In the short term, revisions will usually occur due to acute/chronic infection, instability and/or subject experiencing severe pain due to various causes. This data will be used in combination with clinical assessment, target history / examination and further investigation to determine likelihood of requiring revision.

For all cases where revision was necessary, the investigator must record and forward a description of intraoperative findings including presence of local reaction to implant, gross subsidence of implant and any intraoperative findings relating to the device failure. This information will be recorded by the intraoperative product specialist, and information will be submitted to the TGA via the medical device incident reporting guide. Explant analysis will occur throughout the duration of this study.

### Frequency and plans for auditing trial conduct {23}

On-site monitoring visits shall occur throughout the course of the clinical study by the Chief Investigator. The Chief Investigator shall permit and assist the IDSM (should they choose to monitor the study) to carry out verification of completed case report forms (CRFs) against data in the source documents, which shall occur as per the departmental policy for undertaking such activities.

All personnel involved with the conduct of the study must undertake to maintain the confidentiality of patients in the study. The requirements of the current Good Clinical Practice guidelines will be adhered to for data processing.

### Plans for communicating important protocol amendments to relevant parties (e.g. trial participants, ethical committees) {25}

Annual reports with progress of the study will be submitted to the ethics committee. Any SAE will reported to the committee as per section ‘Adverse event reporting and harms {22}’. Any changes to the protocol will be approved by the St John of God HREC. If there are major changes to protocol, participants will be notified at their follow-up appointment and re-consented to continue in the study.

### Dissemination plans {31a}

The findings of this study will be published in peer-review journals. There are no terms or conditions to the funding that may impact upon publication and dissemination. Authorship will reflect the amount of time spent designing the study, collating the data and writing the manuscript.

## Discussion

This study differs from published randomised control trials in that it is utilising FA rather than KA as the intervention group. FA differs from KA in that the implant positioning is determined by the collateral ligament tension of the knee, and FA has boundaries on the coronal, sagittal and axial alignment of the prosthesis, limiting the very extremes of alignment and preventing outliers. Functional alignment is a new technique and different methods are currently being used. In this study, a KA alignment starting point will be utilised.

The MA cohort of this study will allow up to 2° of coronal plane adjustment. This is equivalent to allowing an adjusted mechanical alignment. Both techniques utilise the extra information provided with RATKA to minimise soft tissue releases. RATKA allows accuracy of bony cuts to accurately implant the prosthesis and give the best representation of the difference between these two alignment techniques.

### Trial status

Study protocol 1.6, 4th of March 2021

Recruitment will begin in April 2021 with completion of recruitment expected by January 31st 2022.

## Supplementary Information

**Additional file 1.** Functional Testing protocols.

## Abbreviations

AE: Adverse event
AOANJRR: Australian Orthopaedic Association National Joint Registry
AP: Anteroposterior
CI: Chief Investigator
CRF: Case Report Form
EQ5D-5 L: European Quality of Life Measurement – 5 Dimension
FA: Functional alignment
FJS: Forgotten Joint Score
HKA: Hip-knee-ankle angle
HREC: Human Research Ethics Committee
IDSM: Independent data safety monitor
KA: Kinematic alignment
KOOS JR: Knee Injury and Osteoarthritis Outcome Score, Joint Replacement version
LAT: Lateral
MA: Mechanical alignment
OA: Osteoarthritis
OKS: Oxford knee score
PHK: Perth Hip & Knee Clinic
PICF: Patient Information and Consent Form
PROMS: Patient-reported outcome measures
QA: Quality assurance
RATKA: Robotically assisted total knee arthroplasty
RCT: Randomised control trial
RP: Research physiotherapist
SAE: Serious adverse event
TKA: Total knee arthroplasty

## Declarations

Ethics approval and consent to participate {24}

HREC approval for this study has been gained from the St John of God Healthcare Human Research Ethics Committee (Perth, Western Australia): approval number 1626. Written, informed consent to participate will be obtained from all participants.

Consent for publication {32}

As part of the consent process for this study, participants consent to the results of this study being published. A model consent form is available upon request if the journal requires this.

## References

[1] de Steiger RN, Liu Y-L, Graves SE (2015). Computer navigation for total knee arthroplasty reduces revision rate for patients less than sixty-five years of age. J Bone Joint Surg Am.

[2] Jorgensen NB, McAuliffe M, Orschulok T, Lorimer MF, de Steiger R (2019). Major aseptic revision following total knee replacement: a study of 478,081 total knee replacements from the Australian Orthopaedic Association National Joint Replacement Registry. J Bone Joint Surg Am.

[3] Bourne RB, Chesworth BM, Davis AM, Mahomed NN, Charron KDJ. Patient satisfaction after total knee arthroplasty: who is satisfied and who is not?, Clinical Orthopaedics and related research®. 2010;468(1):57–63. 10.1007/s11999-009-1119-9 PMC2795819 19844772

[4] Dunbar MJ, Richardson G, Robertsson O. I can’t get no satisfaction after my total knee replacement: rhymes and reasons. Bone Joint Journal. 2013;95-B(11 Suppl A):148–52. 10.1302/0301-620X.95B11.32767 24187375

[5] Lingard EA, Katz JN, Wright EA, Sledge CB (2004). Predicting the outcome of total knee arthroplasty. J Bone Joint Surg.

[6] Gunaratne R, Pratt DN, Banda J, Fick DP, Khan RJK, Robertson BW (2017). Patient dissatisfaction following total knee arthroplasty: a systematic review of the literature. J Arthroplasty.

[7] Bellemans J, Colyn W, Vandenneucker H, Victor J (2012). The Chitranjan Ranawat award: is neutral mechanical alignment normal for all patients? The concept of constitutional varus. Clin Orthop Relat Res.

[8] Patil S, McCauley JC, Pulido P, Colwell CW (2015). How do knee implants perform past the second decade? Nineteen- to 25-year followup of the press-fit condylar design TKA. Clin Orthop Relat Res.

[9] MacDessi SJ, Griffiths-Jones W, Harris IA, Bellemans J, Chen DB (2021). Coronal plane alignment of the knee (CPAK) classification. Bone Joint J.

[10] Rivière C, Iranpour F, Auvinet E, Howell S, Vendittoli PA, Cobb J (2017). Alignment options for total knee arthroplasty: a systematic review. Orthop Traumatol Surg Res.

[11] Vanlommel L, Vanlommel J, Claes S, Bellemans J (2013). Slight undercorrection following total knee arthroplasty results in superior clinical outcomes in varus knees. Knee Surg Sports Traumatol Arthrosc.

[12] Howell SM, Howell SJ, Kuznik KT, Cohen J, Hull ML (2013). Does a kinematically aligned total knee arthroplasty restore function without failure regardless of alignment category?. Clin Orthop Relat Res.

[13] Almaawi AM, Hutt JRB, Masse V, Lavigne M, Vendittoli P-A (2017). The impact of mechanical and restricted kinematic alignment on knee anatomy in total knee arthroplasty. J Arthroplasty.

[14] Hutt JRB, LeBlanc MA, Massé V, Lavigne M, Vendittoli PA (2016). Kinematic TKA using navigation: surgical technique and initial results. Orthop Traumatol Surg Res.

[15] Matsumoto T, Takayama K, Ishida K, Hayashi S, Hashimoto S, Kuroda R (2017). Radiological and clinical comparison of kinematically versus mechanically aligned total knee arthroplasty. Bone Joint J.

[16] Waterson HB, Clement ND, Eyres KS, Mandalia VI, Toms AD (2016). The early outcome of kinematic versus mechanical alignment in total knee arthroplasty: a prospective randomised control trial. Bone Joint J.

[17] Dossett HG, Estrada NA, Swartz GJ, LeFevre GW, Kwasman BG (2014). A randomised controlled trial of kinematically and mechanically aligned total knee replacements: two-year clinical results. Bone Joint J.

[18] Young SW, Walker ML, Bayan A, Briant-Evans T, Pavlou P, Farrington B. The Chitranjan S. Ranawat award: no difference in 2-year functional outcomes using kinematic versus mechanical alignment in TKA: a randomized controlled clinical trial. Clin Orthop Relat Res. 2017;475(1):9–20. 10.1007/s11999-016-4844-x PMC5174030 27113595

[19] Laende EK, Richardson CG, Dunbar MJ (2019). A randomized controlled trial of tibial component migration with kinematic alignment using patient-specific instrumentation versus mechanical alignment using computer-assisted surgery in total knee arthroplasty. Bone Joint J.

[20] MacDessi SJ, Griffiths-Jones W, Chen DB, Griffiths-Jones S, Wood JA, Diwan AD (2020). Restoring the constitutional alignment with a restrictive kinematic protocol improves quantitative soft-tissue balance in total knee arthroplasty: a randomized controlled trial. Bone Joint J.

[21] McEwen PJ, Dlaska CE, Jovanovic IA, Doma K, Brandon BJ (2020). Computer-assisted kinematic and mechanical axis total knee arthroplasty: a prospective randomized controlled trial of bilateral simultaneous surgery. J Arthroplast.

[22] MacDessi SJ, Gharaibeh MA, Harris IA (2019). How accurately can soft tissue balance be determined in total knee arthroplasty?. J Arthroplast.

[23] Levine BR, Springer BD, Golladay GJ (2020). Highlights of the 2019 American joint replacement registry annual report. Arthroplasty today.

[24] Kayani B, Konan S, Ayuob A, Onochie E, Al-Jabri T, Haddad FS (2019). Robotic technology in total knee arthroplasty: a systematic review. EFORT Open Rev.

[25] Chang JS, Kayani B, Wallace C, Haddad FS (2021). Functional alignment achieves soft tissue balance in total knee arthroplasty as measured with quantitative sensor-guided technology. Bone Joint J.

[26] Oussedik S, Abdel MP, Victor J, Pagnano MW, Haddad FS (2020). Alignment in total knee arthroplasty. Bone Joint J.

[27] Kayani B, Konan S, Tahmassebi J, Oussedik S, Moriarty PD, Haddad FS (2020). A prospective double-blinded randomised control trial comparing robotic arm-assisted functionally aligned total knee arthroplasty versus robotic arm-assisted mechanically aligned total knee arthroplasty. Trials..

[28] Young SW. ANZCTR - CAMELOT Study. Accessed 26th February 2021. Available from: http://www.anzctr.org.au/Trial/Registration/TrialReview.aspx?id=378892&isReview=true.

[29] MacDessi SJ. ANZCTR - RASKAL Study. Accessed 26th February 2021. Available from: http://www.anzctr.org.au/Trial/Registration/TrialReview.aspx?id=381170&isReview=true.

[30] Akagi M, Oh M, Nonaka T, Tsujimoto H, Asano T, Hamanishi C (2004). An anteroposterior axis of the tibia for total knee arthroplasty. Clin Orthop Relat Res.

[31] Garratt AM, Brealey S, Gillespie WJ, Team DT (2004). Patient-assessed health instruments for the knee: a structured review. Rheumatology (Oxford, England).

[32] Kreibich DN, Vaz M, Bourne RB, Rorabeck CH, Kim P, Hardie R (1996). What is the best way of assessing outcome after total knee replacement?. Clin Orthop Relat Res.

[33] Ganko A, Engebretsen L, Ozer H (2000). The Rolimeter: a new arthrometer compared with the KT-1000. Knee Surg Sports Traumatol Arthroscopy.

[34] Burns SP, Spanier DE (2005). Break-technique handheld dynamometry: relation between angular velocity and strength measurements. Arch Phys Med Rehabil.

[35] Lu TW, Hsu HC, Chang LY, Chen HL. Enhancing the examiner's resisting force improves the reliability of manual muscle strength measurements: comparison of a new device with hand-held dynamometry. J Rehabil Med. 2007;39(9):679–84. 10.2340/16501977-0107. 10.2340/16501977-0107 17999004

[36] O’Shea SD, Taylor NF, Paratz JD (2007). Measuring muscle strength for people with chronic obstructive pulmonary disease: retest reliability of hand-held dynamometry. Arch Phys Med Rehabil.

[37] Holtz N, Hamilton DF, Giesinger JM, Jost B, Giesinger K (2020). Minimal important differences for the WOMAC osteoarthritis index and the forgotten joint Score-12 in total knee arthroplasty patients. Bmc Musculoskelet Di.

[38] Clement ND, Scott CEH, Hamilton DF, MacDonald D, Howie CR (2021). Meaningful values in the forgotten joint score after total knee arthroplasty: minimal clinical important difference, minimal important and detectable changes, and patient-acceptable symptom state. Bone Jt J.

[39] Fox J. Applied regression analysis, linear models, and related methods. Third Edition. Sage; 2016. Los Angeles.

