# Supplementary material for: A prospective randomised control trial comparing functional with mechanical axis alignment in total knee arthroplasty: study protocol for an investigator initiated trial
Source: Trials. 2021 Aug 9;22:523. doi: 10.1186/s13063-021-05433-z (PMC8351154; doi:10.1186/s13063-021-05433-z)

**Additional file 1**

**Functional Testing protocols**

**30-second Chair Stand Test** (32,37)

Participant

- Comfortable walking footwear (e.g. tennis shoes/cross trainers) should be worn.
- The participant sits in the chair in a position that allows them to place their feet flat on the floor, shoulder width apart, with knees flexed slightly more than 90 degrees so that their heels are somewhat closer to the chair than the back of their knees.
- The arms are crossed at the wrists and held close to the chest (across chest).

Tester

•The tester stands close to the side of the chair for safety and so as they can observe the technique, ensure that the participant comes to a full stand and full sit position during the test.

Practice

•A practice trial of one or two slow paced repetitions is recommended before testing to check technique and understanding.

Procedure

- From the sitting position, the participant stands up completely up so hips and knees are fully extended, then completely back down, so that the bottom fully touches the seat. This is repeated for 30 seconds.
- Same chair should be used for re-testing within site.
- If the person cannot stand even once then allow the hands to be placed on their legs or use their regular mobility aid. This is then scored as an adapted test score.

Equipment

- Timer / stopwatch
- Straight back chair with a 17 inch seat height, preferably without arms
  - The same chair should be used for re-testing between sites.

Verbal instructions – to be followed exactly.

“For this test, do the best you can by going as fast as you can but don’t push yourself to a point of overexertion or beyond what you think is safe for you.

1. Place your hands on the opposite shoulder so that your arms are crossed at the wrists and held close across your chest. Keep your arms in this position for the test.
2. Keep your feet flat on the floor and at shoulder width apart.
3. On the signal to begin, stand up to a full stand position and then sit back down again so as your bottom fully touches the seat.
4. Keep going for 30 seconds and until I say stop.
5. Get ready and START”.

Scoring

- On the signal to begin, start the stop watch. Count the total number of chair stands (up and down equals one stand) completed in 30 seconds. If a full stand has been completed at 30 seconds (i.e., standing fully erect or on the way down to the sitting position), then this final stand is counted in the total.
- The participant can stop and rest if they become tired. The time keeps going.
- If a person cannot stand even once then the score for the test is zero.
- Next, allow the hands to be placed on their legs or use their regular mobility aid. If the person can stand with adaptions, then record the number of stands as an adapted test score (see score sheet). Indicate the adaptations made to the test.

N.B. The individual should use the assistive device (if any) they would normally use to perform the activity at the time of testing, irrespective of how they performed it previously. However, if an assistive device/rail is used, then it should be recorded for that occasion.

**Dynamometer Testing**

Before testing, subjects to be seated in the exercise chair or plinth with no back support. The patient to be instructed to remain seated in an upright position and place both hands on his or her upper legs to avoid compensation. The "make" method for strength testing to performed rather than the "break" method as it has been shown to have better reliability and provide more accurate measures.

For knee flexion, dynamometer to be placed on the posterior aspect of the calcaneus. For knee extension, the dynamometer to be positioned perpendicular to the anterior aspect of the tibia, 5 cm proximal of the medial malleolus. Patient limb to be positioned at 20 and 90 degrees of flexion, measured with a goniometer, in an open chain position. As previous studies have experienced patients being too strong for testers, the dynamometer will be fixated to the base of the plinth via a purpose built cradle during testing. At each angle, patients may have one test attempt prior to the recording attempt. The patient to be instructed to gradually build up strength for two seconds to avoid explosive contraction, then to continue with a three-second maximal contraction as used in previous studies.

Patient to be instructed identically each time, and encouraged to “go go go” during each attempt. There is to be a 30 second rest between each attempt to allow for muscle recovery. Knee extension is be performed first, followed by knee flexion. The initial measurement is to be performed on the unaffected leg, followed by the affected leg, and thereafter alternated in a similar fashion for both flexion and extension. This will be measured three times of each leg.


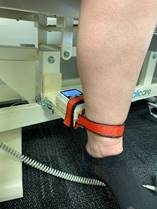

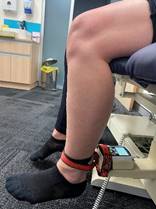


**Figure 1**- fixed dynamometer testing in flexion

**Lachmeter Testing**

To be performed at 20* and 90* knee flexion. Plinth to be set up to correct angles.

**Figure 2**
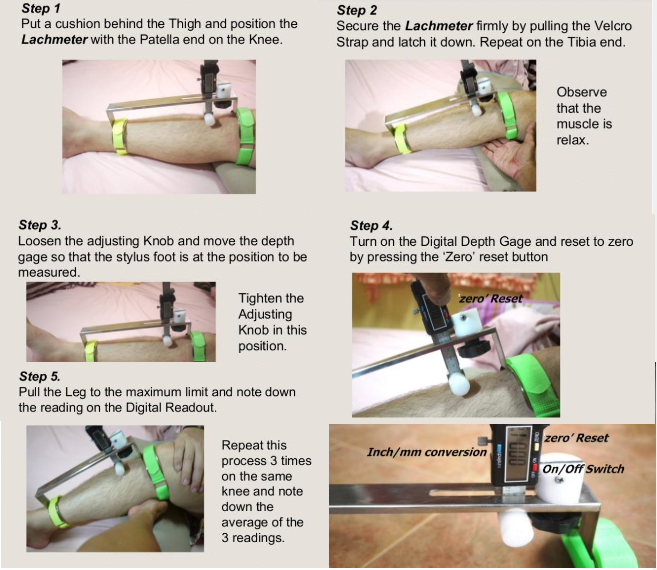

Supplement: Supplementary file 1 — Additional file 1. Functional Testing protocols. [file 13063_2021_5433_MOESM1_ESM.docx]
